# Fluorescent and Electron-Dense Green Color Emitting Nanodiamonds for Single-Cell Correlative Microscopy

**DOI:** 10.3390/molecules25245897

**Published:** 2020-12-13

**Authors:** Neeraj Prabhakar, Markus Peurla, Olga Shenderova, Jessica M. Rosenholm

**Affiliations:** 1 Pharmaceutical Sciences Laboratory, Faculty of Science and Engineering, Åbo Akademi University, 20520 Turku, Finland; jerosenh@abo.fi; 2 Institute of Biomedicine, Faculty of Medicine, University of Turku, 20520 Turku, Finland; markus.peurla@utu.fi; 3 Cancer Research Laboratory FICAN West, Institute of Biomedicine, University of Turku, 20520 Turku, Finland; 4 Turku Bioscience Centre, University of Turku and Åbo Akademi University, 20520 Turku, Finland; 5 Adámas Nanotechnologies, Inc., 8100 Brownleigh Drive, Suite 120, Raleigh, NC 27617, USA; oshenderova@adamasnano.com

**Keywords:** CLEM, nanodiamonds, single-cell microscopy, fiducial, dual probes

## Abstract

Correlative light and electron microscopy (CLEM) is revolutionizing how cell samples are studied. CLEM provides a combination of the molecular and ultrastructural information about a cell. For the execution of CLEM experiments, multimodal fiducial landmarks are applied to precisely overlay light and electron microscopy images. Currently applied fiducials such as quantum dots and organic dye-labeled nanoparticles can be irreversibly quenched by electron beam exposure during electron microscopy. Generally, the sample is therefore investigated with a light microscope first and later with an electron microscope. A versatile fiducial landmark should offer to switch back from electron microscopy to light microscopy while preserving its fluorescent properties. Here, we evaluated green fluorescent and electron dense nanodiamonds for the execution of CLEM experiments and precisely correlated light microscopy and electron microscopy images. We demonstrated that green color emitting fluorescent nanodiamonds withstand electron beam exposure, harsh chemical treatments, heavy metal straining, and, importantly, their fluorescent properties remained intact for light microscopy.

## 1. Introduction

Correlative light and electron microscopy (CLEM) is gaining popularity as a microscopy technique to study functional and ultrastructural aspects of a cell [1,2,3,4,5,6,7,8,9]. CLEM is enabling researchers to combine the strengths of light and electron microscopes, and subsequently limiting the shortcomings with the respective technique. CLEM combines the power of both techniques and opens up the opportunity to visualize rare events in cells. CLEM is usually performed to study cells under living or fixed conditions with light microscopy (LM). LM is used to rapidly visualize a large field of view to locate fluorescent molecules of interest in cells [3,10]. Light microscopy (LM) allows the selection of regions of interest in cells for further investigation. Subsequently, the same cell is investigated to provide corresponding ultrastructural information at a much-improved resolution with a transmission electron microscopy (TEM) or scanning electron microscopy (SEM). There are numerous methods developed to successfully perform CLEM experiments [1,4,6,11,12,13]. These developed methods have applied the latest technical improvements of light and electron microscopes. One of the recent focuses of CLEM method development lies with obtaining an improved resolution with LM to bridge the existing resolution gap with electron microscopy (EM). The traditional LM is restricted in resolution due to the diffraction limit [14,15,16]. Some CLEM methods have emphasized the application of super-resolution light microscopes to overcome the latter [17,18,19,20,21,22,23,24,25]. Moreover, on the electron microscopy front, techniques such as cryo-electron microscopy (cryo-EM) and automated serial sectioning were employed to obtain the structure preservation and sectioning of the whole-cell volume [24,26,27]. Thus, maximizing the technical capability to obtain cellular information in their native state by cryo-EM [11,25,28,29], while automated serial sectioning by scanning electron microscopy (SEM) provides information across all three dimensions of a cell [30,31,32,33].

Successful execution of CLEM experiments requires a series of steps, including suitable subcellular labeling, sample processing, multimodal imaging, and precise image correlation. The multimodal imaging platforms require common landmarks to correlate cellular features. In most existing CLEM methods, the multimodal (LM and EM) image is correlated by externally applying fluorescent beads (100 nm) [34]; gold nanoparticles attached with organic dyes [11,35,36] or quantum dots (QDs) as fiducials [37,38]. The limitations of using organic dyes and QDs as fiducial markers are the instability of photobleaching and electron beam exposure [39,40,41]. These fiducials can be irreversibly quenched after exposure to the electron beam generated during electron microscopy [42,43]. Hence, these fiducials are not robust for successive LM after electron beam exposure. Other practical limitations of externally applied fiducials are the improper distribution of fluorescent beads on the target sample and potential cytotoxicity concerns with QDs [44,45,46]. These current limitations with applied fiducials for image correlation can be addressed by substituting it with an intracellular fluorescent and electron dense landmark that offers chemical robustness, optical photostability, and potential for immunolabeling. Attachment of antibodies to fiducials would greatly enhance the applicability for targeted imaging of biomolecules in CLEM experiments.

Fluorescent nanodiamonds (FNDs) are unique carbon-based nanomaterials that are both fluorescent and electron-dense [19,47,48,49,50] and thus, detectable with LM and EM [19,51,52,53]. They are well studied for their unique photostability [54,55,56,57,58]. The origin of fluorescence lies in the complexes formed by vacancies and impurities in a diamond crystal [59,60,61]. These unique complexes are known as color centers in FNDs. FNDs are well compatible with multimodal imaging techniques such as stimulated emission depletion microscopy (STED) [19,50,62,63], two-photon microscopy [64,65], photoacoustic microscopy [66,67], and live-cell fluorescence microscopy [19]. The notable color centers are those emitting in blue, green, yellow, red, and near-infrared spectral regions [68,69,70]. For cell imaging, FNDs are biocompatible, non-toxic, and can be internalized efficiently in the cell via endocytosis [47,71,72,73,74,75]. The FNDs are compatible with immunolabeling [76,77,78,79]. FNDs are applied for direct targeting of a biological molecule by attaching a standard antibody using a streptavidin conjugation. These attractive multicolor optical bioimaging [80] and along the electron-dense nature allows detectability with electron microscopy make them suitable for application in CLEM experiments [19,51,81,82]. Recently several attempts were made to utilize only red color emitting FNDs for CLEM [19,51,52,81,82,83]. H-C Chang et al., 2018, demonstrated the application of biotinylated lipids encapsulated red FNDs for subdiffraction imaging of antigens on the cell surface with correlative light-electron microscopy (CLEM) with a scanning electron microscopy [52]. Han et al. 2019, demonstrated the individual red FNDs were directly visualized by energy-filtered transmission electron microscopy [82]. We have previously reported the application red fluorescent FNDs for super resolution CLEM [19].

However, our CLEM application is based on green color emissive FNDs. Green color FNDs are structurally and chemically similar to red FNDs, whereas optical properties are different. In this paper, we demonstrate the potential of green color emitting FNDs (green FNDs) as robust fiducials for single-cell correlative microscopy. We have generated a workflow by performing TEM imaging of intracellular green FNDs and successive LM over the electron beam exposed 100 nm thin section. The TEM and LM images were precisely correlated using green FND landmarks.

## 2. Results

### 2.1. TEM of Green FNDs in Cells

**Figure 1:**
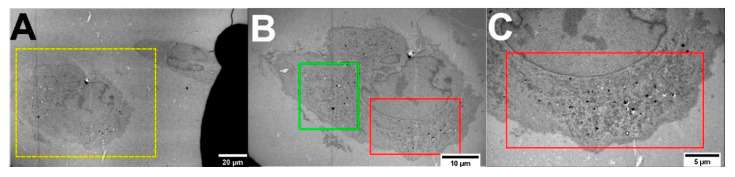
TEM imaging of green fluorescent nanodiamonds (FNDs) internalized in MDA-MB-231 cells. (**A**) A 100 nm thin cell section was placed over a marked electron microscopy (EM) grid that allowed the identification of the cell for correlation. Yellow box showing the cell of interest. (**B**) The cell of interest with two regions of interest (ROIs; green and red boxes) selected for image correlation with the corresponding light microscopy (LM) ROIs. (**C**) A high magnification TEM image of ROI (red box) showing the internalized green FNDs (dark spots) landmarks.

A 100 nm thin TEM sections of green FND internalized MDA-MB-231 cells were prepared for TEM imaging. The section was placed over a formvar-coated marked EM grid to select the cell for correlative TEM and LM (Figure 1). The cell of interest for the image correlation was selected in Figure 1A. The low magnification TEM image in Figure 1A shows the cell of interest (yellow square). The green FNDs are visible as dark spots in Figure 1B. In this Figure (Figure 1B), two regions of interest (ROIs) are marked by the green and red squares. These ROIs were later used to correlate with the corresponding ROIs from LM images. In Figure 1C, a high magnification TEM image shows green FNDs localized within vesicular space. FNDs have a general tendency to localize in an aggregated manner in cellular vesicles (Figure S1A,B) [47,63].

### 2.2. LM of Green FNDs

**Figure 2:**
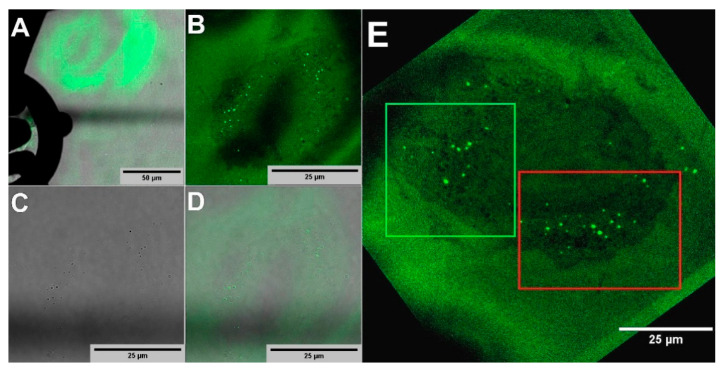
LM performed over a 100 nm thick section on the TEM grid. (**A**) Low magnification overview of the cell of interest located over the TEM grid. (**B**) LM of the cell of interest (rotated view) that showed the fluorescence from green FNDs is localized in a few spots. The sample generated an extremely high background signal, which also facilitated the visualization of the cell of interest. (**C**) Dark spots of green FNDs can be even visualized in the bright-field image. (**D**) Overlay of B&C. (**E**) Maximum intensity z-projection of the green FNDs signal with two corresponding ROIs (green and red boxes), selected for image correlation with respective TEM ROIs. The image is aligned to match with the corresponding TEM ROIs (Figure 1C).

To evaluate the robustness of green FNDs for CLEM experiments, we demonstrated here that green FNDs remained fluorescently stable even after TEM imaging at 80 kV acceleration voltage and survive harsh chemical treatment during sample preparation for TEM. Figure 2 shows fluorescence imaging of the same region as Figure 1. In Figure 2A, a low magnification overlay image of fluorescence and bright field channels shows the marked EM grid with letters and autofluorescence originating from the EM section. In Figure 2B, the confocal image of the EM section of the cell of interest with green FNDs (green dots) is shown. The green FNDs are seen aggregated and confined to a few spots in the cell of interest. The fluorescence signal of individual green FNDs can be exceptionally low in comparison to the high background autofluorescence signal due to glutaraldehyde fixation and heavy metal staining, makes it challenging to detect smaller aggregates precisely. Vesicle aggregated green FNDs provide a high local green FND concentration and stronger fluorescence signal for easier detection. Consequently, green FNDs aggregation is ideal for simplification of the image correlation between the LM and TEM images. Aggregated green FNDs can be visualized as dark spots even in a bright-field image (Figure 2C). The overlay of fluorescence and bight field channels is shown in Figure 2D. In Figure 2E the maximum intensity Z-projection image of green FNDs is shown with two ROIs (green and red boxes) corresponding to those in the TEM image in Figure 1C.

### 2.3. Correlation between TEM and LM Images

**Figure 3:**
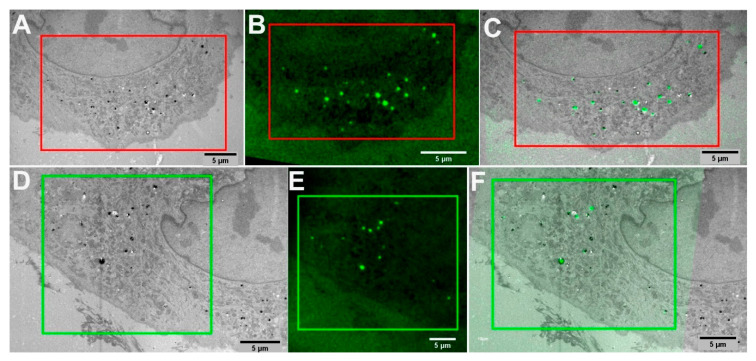
Image correlation facilitated by green FNDs for two different (green and red) ROIs of the cell of interest. (**A**) TEM image were intracellular green FNDs can be seen in ROI (red box). (**B**) LM image of the green FNDs (red box). (**C**) Correlated image of ROI (red box) aligned by using common green FNDs landmarks present in both TEM (**A**) and LM (**B**) images. (**D**) TEM image of intracellular green FNDs can be seen in ROI (green box). (**E**) LM of green FNDs (green box). (**F**) Correlated image of ROI (green box) aligned by using common green FNDs landmarks present in both TEM (**D**) and LM Image (**E**) images.

The green FNDs based CLEM method for multimodal image correlation was performed with a dedicated software plugin “eC-CLEM” [84]. The LM images were preprocessed to enhance brightness and contrast with ImageJ, and prealigned to match orientation with TEM images. The efficiency and precision of a non-rigid image correlation can be enhanced by maximizing the number of fiducial landmarks available for correlation with both modalities. We used 10–19 FNDs fiducial landmarks in our CLEM experiments, which resulted in a highly precise image correlation. In Figure 3A,B, the corresponding green FNDs (red boxes) from LM and TEM were matched by selecting individual green FNDs in the automatic mode of eC-CLEM. In Figure 3C, the overlay of correlated LM and TEM images is shown. The ROIs (green and red boxes) from TEM (Figure 1B) and LM (Figure 2E) were matched point-by-point using green FNDs as fiducials to generate a highly precise overlay. Similarly, ROI (green boxes) in Figure 3D,E were matched using common green FNDs as landmarks. The results of the image correlation of Figure 3D,E is shown in Figure 3F.

## 3. Discussion

We demonstrated that green FNDs could facilitate intracellular image correlation by accurately overlaying LM and TEM images. The green FNDs are potent landmarks in CLEM experiments, which can be easily detected with both LM and TEM. The presented method is straightforward to perform within regular microscopy facilities. We also showed that green FNDs were resistant to degradation under electron beam exposure and fluorescence properties remained intact upon chemical treatments used in TEM sample processing. The robustness of green FNDs allowed them to be imaged by electron exposure with EM and successive imaging with a fluorescence microscope. This advantage can potentially be used in immunoelectron microscopy on EM grids (e.g., Tokuyasu method). The chemically active surface of green FNDs is versatile and known to be suitable for the attachment or delivery of biomolecules [53,79,80,82,83]. In such a case, antibody conjugated green FNDs could recognize the molecule of interest and enable detection with an EM. Furthermore, the fluorescence stability of green FNDs would allow imaging with a fluorescence microscope. Therefore, in the future, antibody conjugated green FNDs would be a well-suited immunolabeling method for performing correlative light and electron microscopy. Moreover, dual antigen detection can be performed by using two distinct color FNDs (red and green), conjugated with two antibodies. Furthermore, the green FNDs could enable 3-dimensional CLEM imaging methods, where the 3D information from both electron microscopy (serial sectioning) and fluorescence microscopy (volume stack) can be combined for investigating the complex cellular processes across the full volume of a cell. 

## 4. Materials and Methods

### 4.1. Green Fluorescent Nanodiamonds (Green FNDs)

Adámas Nanotechnologies (Raleigh, NC, USA) produced the green FNDs used in the study. The detailed material synthesis, characterization and optical properties are described in Dei Cas et al., 2019 [85] and Nunn et al., 2019 [68]. FNDs with green fluorescence emission were produced from synthetic type Ib high-pressure high temperature (HPHT) nanodiamond particles with an initial substitutional nitrogen content of 100 ppm and average particle size of 100 nm. Particles were irradiated with high energy electrons (3 MeV) to generate vacancies and subsequently annealed using previously reported[85] rapid thermal annealing approach at 1800 °C for 2 min in a hydrogen atmosphere. Subsequent oxidation in a mixture of nitric/sulfuric acids was used to remove graphitic carbon and provide a carboxylated (-COOH) terminal surface chemistry on the particles.

### 4.2. Cell Culture

For the experiments, MDA-MB-231 cells, (human breast adenocarcinoma collected from Turku Biosciences, University of Turku) were cultured in Dulbecco’s modified Eagle’s medium (DMEM) supplemented with 10% fetal bovine serum, 2 mM L-glutamine, and 1% penicillin-streptomycin (*v*/*v*). Of green FNDs particles 10 µg/mL were prepared in 1 mL of cell growth media. Then, the growth media with particles was added to the cells. The cells were allowed to incubate with FNDs for 24 h [47].

### 4.3. Transmission Electron Microscopy (TEM)

The cell was fixed with 5% glutaraldehyde s-collidine buffer, postfixed with 1% OsO4 containing 2.5% potassium ferrocyanide, dehydrated with ethanol, and flat embedded in a 45,359 Fluka Epoxy Embedding Medium kit. Thin sections were cut using an ultramicrotome to a thickness of 100 nm. The sections were stained using uranyl acetate and lead citrate to enable detection with TEM. The section was mounted on marked EM grids. The section was examined using a JEOL JEM-1400 Plus transmission electron microscope operated at 80 kV acceleration voltage [47].

### 4.4. Confocal Microscopy

The marked EM grid was imaged with Leica TCS SP8 confocal microscope (Leica Microsystems, Wetzlar, Germany), using a 100× oil objective. Green FNDs were excited by a 488 nm white light laser (WLL). Fluorescence emission collected at 510–550 nm with PMTs (photomultiplier tubes) for green FNDs. 

### 4.5. Image Correlation

The raw images from FM and TEM were preprocessed to enhance brightness and contrast with ImageJ. The multimodal datasets were registered using the eC-CLEM plugin, a free open-source software plugin, in the advanced usage mode on the Icy bioimage analysis platform [84]. To match the large datasets on a desktop (i7, 16 Gb RAM), the EM stack was binned 4 times. In the advanced usage mode, the eC-CLEM software also evaluated the need to apply non-rigid registration to obtain accurate registration. The FM stack was matched to the binned dataset using the green FNDs as landmarks, targeting the center of the green FNDs aggregates both in LM and EM using orthogonal views from Icy. From 10 to 17 green FNDs aggregates were enough to achieve good overlay accuracy. The weighing of each landmark operated by eC-CLEM compensate for the shifts observed between the LM and the EM dataset and rigid registration leads to an accurate full registration. Non-rigid registrations were performed, to generate the final overlay, the transformation was applied to the LM dataset to match the original EM dataset using the “apply a reduced scaled transform to a full-size image” function from eC-CLEM (Advanced usage).

## Supplementary Materials

The following are available online, Figure S1: FNDs have a general tendency to localize in an aggregated manner.
