# Supplementary material for: Fluorescent and Electron-Dense Green Color Emitting Nanodiamonds for Single-Cell Correlative Microscopy
Source: Molecules. 2020 Dec 13;25(24):5897. doi: 10.3390/molecules25245897 (PMC7764487; doi:10.3390/molecules25245897)
Supplement: Supplementary file 1 [file molecules-25-05897-s001.pdf]

Fluorescent and electron-dense green color emitting nanodiamonds for single-cell correlative microscopy

Neeraj Prabhakar[1], Markus Peurla[2], Olga Shenderova[3] and Jessica M. Rosenholm[1]*

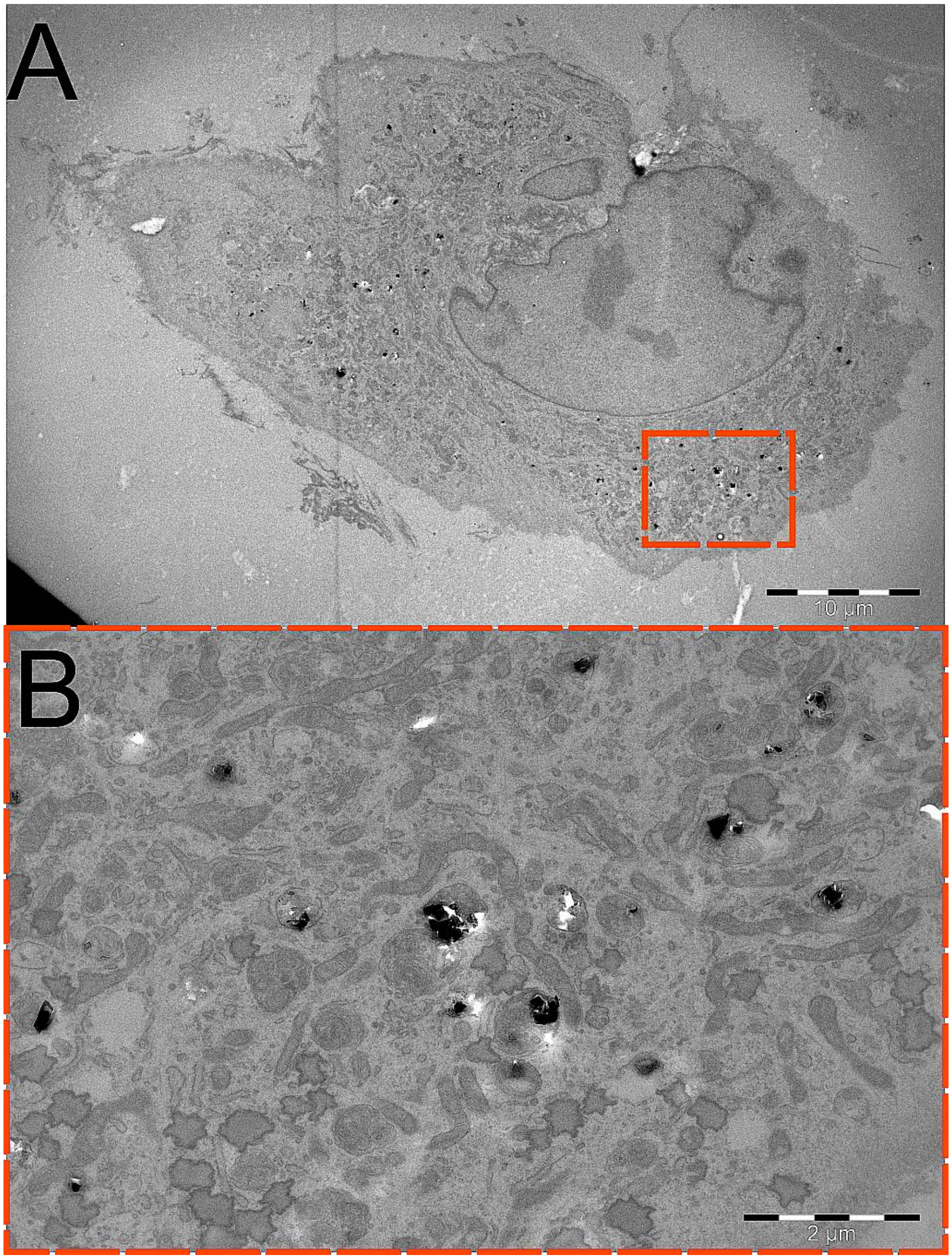

Figure S1. A) FNDs have a general tendency to localize in an aggregated manner. Oragne box shows the intracellular gFNDs. B) A high-resolution image of gFNDs shows the intracellular aggregation (Orange box).
